# Effect of market participation on the food and nutrition security status of the rural smallholder farmers: the case of Limpopo and Mpumalanga provinces, South Africa

**DOI:** 10.3389/fsufs.2023.1097465

**Published:** 2023-11-16

**Authors:** Simphiwe Innocentia Hlatshwayo, Temitope Oluwaseun Ojo, Mjabuliseni Simon Cloapas Ngidi

**Affiliations:** 1 African Center for Food Security, School of Agricultural, Earth and Environmental Sciences, College of Agriculture, Engineering and Science, University of KwaZulu-Natal, Pietermaritzburg, South Africa; 2 Center for Transformative Agricultural and Food Systems, School of Agricultural, Earth and Environmental Sciences, College of Agriculture, Engineering and Science, University of KwaZulu-Natal, Pietermaritzburg, South Africa; 3 Department of Agricultural Economics, Obafemi Awolowo University, Ile-Ife, Nigeria; 4 Disaster Management Training and Education Center for Africa, University of the Free State, Bloemfontein, South Africa; 5 Department of Agricultural Extension and Rural Resource Management, School of Agricultural, Earth and Environmental Sciences, College of Agriculture, Engineering and Science, University of KwaZulu-Natal, Pietermaritzburg, South Africa

**Keywords:** nutrition security, market participation, smallholder farmers, Poisson endogenous treatment effect model, food security

## Abstract

**Introduction:**

Hunger and malnutrition remain serious issues in developing countries, particularly in rural regions. Increased market participation of smallholder farmers can result in improved livelihood and nutrition outcomes. However, smallholder farmers encounter several obstacles that hinder their ability to participate in the market. As a result, the objective of this study is to investigate the factors that influence market participation and its impact on household nutrition security.

**Methods:**

The study relied on secondary data gathered from a sample size of 1,520 people. About 389 of smallholder farmers participated in the market.

**Results and Discussion:**

The results from Food Consumption Score (FSC) cut-off points showed that in the overall sample households, 54% were within the acceptable food consumption diets (>35), while 30 and 16% were in the borderline (21.5–35) and poor diets (0–21), respectively. According to the Household Dietary Diversity Score (HDDS) findings, 57% of smallholder farmers in the total population sample consumed highly diverse diets (consisting of at least six food groups), while 25 and 18% of smallholder farmers consumed diets with medium dietary diversity (consisting of 4–5 food groups) and low dietary diversity (consisting of at most three food groups), respectively. The results from marginal analysis showed that gender of household head, receiving social grants and higher wealth index had a positive impact on market participation. The results from Poisson endogenous treatment effect model showed that household size, ownership of livestock, social grant, wealth index, access to market information, and involvement in crop production had a positive and statistically significant impact on household nutrition security. On the other hand, agricultural assistance showed a negative and significant impact on household nutrition security.

**Conclusion and Recommendations:**

It can be concluded that an improvement in agricultural assistance can improve the household nutrition security status. The improvement of agricultural assistance is more associated with improvement of extension services, which can lead to more production of diverse crops and more market participation. Health extension workers need to do more nutrition programs and workshops in rural areas. These programs and workshops will be intended on providing nutrition education, which will create awareness to smallholder farmers on diverse and balanced food items they should produce, sell, and consume.

## 1 Introduction

Hunger and malnutrition are multifaceted global issues. Despite recent advances in food and nutrition security, the prevalence of malnutrition remains high, particularly in developing countries (Sibhatu et al., 2015). In 2021, world hunger and malnutrition rose to 828 million people [United Nations International Children’s Emergency Funds (UNICEF), 2022]. According to Statistics South Africa (Stats SA) (2022) about 2.1 million (11.6%) of South African households reported experiencing hunger in 2021. The lack of essential vitamins and minerals in most Southern African households causes nutrient malnutrition (Akombi et al., 2017; Drammeh et al., 2019; Adeyeye et al., 2021). Almost 2 billion people are malnourished due to inadequate intakes of vitamins and minerals such as iron and zinc [International Food Policy Research Institute (FPRI), 2014]. Nutritional deficiencies have a significant health impact in terms of lost productivity, impaired physical and mental development, susceptibility to various diseases, and premature death. Malnutrition is most common in women and children (Sibhatu et al., 2015). Previous studies have shown that malnutrition puts children at risk of contracting infectious diseases, stunts growth increases the severity of infections, reduces school and work performance, and kills children under the age of 5 (Baird et al., 2016; Getahun and Fetene, 2021). Furthermore, the studies revealed that pregnant and lactating women are more vulnerable to malnutrition because they oversee their own and their children’s diets. This demonstrates the importance of investing more in nutrition because it can improve household food security.

Nutrient deficiency, malnutrition, and a lack of dietary diversity are very common among rural smallholder farmers because they rely primarily on a few starchy staple food sources and have limited market access (Hirvonen, 2016). Smallholder farmers work in remote areas with limited infrastructure, transportation, and access to capital, technology, and knowledge. The poor functioning of rural markets encourages farmers to produce primarily for their own consumption rather than for sale (Bellon et al., 2016). This means that the majority of smallholders rely on their production to meet their dietary diversity needs, which is insufficient. Farmers who have market access can meet their dietary diversity needs while also generating income. According to Getahun and Fetene (2021), improved market access can lead to smallholder farmers producing high-value agricultural products, giving them a competitive advantage and allowing them to earn a high-expected income. Markets enable farm production to contribute to poverty reduction through income generated from farm produce sales (Ssajakambwe et al., 2020). Markets also drive production because they provide farmers with an incentive to strive to meet buyer demands for quality and quantity (Obi et al., 2012). There is a need to emphasize the role of market access in improving nutrition because it promotes equal distribution of foods and incomes and allows smallholder farmers to access more foods than they produce (Ssajakambwe et al., 2020).

As a result, it is critical to conduct empirical research on the fundamental relationship between market participation and nutrition security (dietary diversity). Understanding the links between household participation in agricultural output markets and dietary diversity could thus help inform nutrition interventions on how agricultural commercialization and rural markets can be leveraged to improve nutrition outcomes among rural smallholder farmers. Despite the critical role that market participation plays in improving the nutrition security of smallholder farmers, there is little evidence-based information linking the two, particularly in South Africa. Mulenga et al. (2021) investigated how participation in output markets affects the dietary diversity of Zambian rural smallholder farmers. Ssajakambwe et al. (2020) investigated the relationship between market access and nutritional security, as well as the factors influencing farmers’ market access and improved nutrition among Ugandan smallholder maize farmers. Lenjiso et al. (2016) studied the impact of smallholder milk market participation on household and intra-household dietary diversity, as well as Ethiopian young children’s nutritional status. These studies were conducted in other parts of Africa, and they demonstrate a gap in linking market participation and nutrition security in South Africa. In this context, the study seeks to (1) determine the factors that impact smallholder farmers’ market participation, and (2) quantify the effect of market participation on rural households’ nutrition security status in two South African provinces.

## 2 Materials and methods

### 2.1 Description of study area

**Figure 1:**
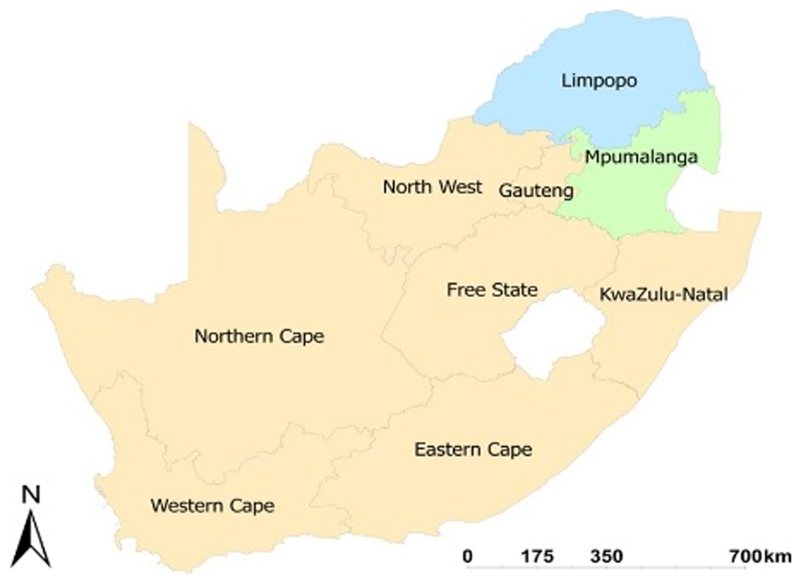
Map of South Africa showing the two different provinces (Mpumalanga and Limpopo) used in this study. Source: http://www.demarcation.org.za/.

The purpose of this study was to determine the impact of market participation on the food and nutrition security of smallholder farmers that are producing crops in Mpumalanga and Limpopo provinces. The study relied on secondary data collected from South Africa’s two provinces (Mpumalanga and Limpopo) in 2016/2017 (refer to Figure 1). Limpopo province has a population of approximately 5.7 million people, with more than 80% of the population living in rural areas (Statistics South Africa, 2015). When compared to other provinces, the province is said to have a high population growth rate (De Cock et al., 2013). Poverty, malnutrition, and food insecurity plague the province as a result of its rapid growth. According to empirical evidence, most households in the province rely on agricultural activities to maintain food security and livelihoods (Manyamba et al., 2012).

Mpumalanga province has a population of 4,128,000 people, accounting for 7.8% of South Africa’s total population (NAFCOC, 2014). The province had the country’s third highest unemployment rate among the nine provinces, and its poverty rate of 39.4% was higher than the national rate (Mpumalanga Department of Finance, 2013). The majority of Mpumalanga’s population, including the majority of the poor, lives in areas of low economic activity. Mpumalanga Province’s vegetation is classified as forest, savannah, and grassland. Mpumalanga agriculture consists of livestock production, sugar production, and crops such as fruits, potatoes, sunflowers, maize, wheat, and nuts (Mpumalanga Department of Finance, 2013).

### 2.2 Data collection method

The study collected data through questionnaires using a quantitative research method. The sample size was chosen at random using the multi-stage stratified random sampling technique. The sample size determination was calculated using statistical software on the 95% confidence interval and 5% margin which each smallholder farmer having an equal chance to being selected. A total of 1,520 participants were chosen from two provinces (Limpopo and Mpumalanga). The quantitative data were collected in four Mpumalanga districts and three Limpopo districts. To maximize effectiveness and avoid selection bias, participants were divided into strata based on similar variables such as socio-demographics, technical, and institutional factors. Farmers, who are crop producers, were questioned on their agricultural production system as well as nutrition security indicators. In the 2016/2017 season, the South African Vulnerability Assessment Committee (SAVAC), led by the Secretariat hosted in the Department of Agriculture, Land Reform, and Rural Development (DALRRD), collected secondary data (available at: www.drdlr.gov.za accessed on June 20, 2020).

### 2.3 Data analysis

The quantitative data were analyzed using Statistical Software for Social Sciences (SPSS) version 24 and STATA statistical software (version 13). The descriptive statistics were obtained to provide the key socioeconomic characteristics of the sampled smallholders and compare how they differed in terms of nutrition security between market participants and non-market participants. The study used internationally recognized food and nutrition security measurement tools to assess the food and nutrition status of smallholder farmers. This included the Household Dietary Diversity Score (HDDS) and Food Consumption Score (FCS).

The HDDS displays the various types of food and the amount of dietary diversity available to a household (Kennedy et al., 2011). Households were asked about the food groups they consumed in the past 24 h in order to determine dietary diversity among households. Swindale and Bilinsky (2006) identified 12 standard food groups that households can consume to improve their nutritional status, including (1) milk and milk products, (2) meat, (3) pulses/legumes/nuts, (4) roots and tubers, (5) poultry and eggs, (6) cereal, (7) fish and seafood, (8) oil/fats, (9) sugar/honey, (10) vegetables, (11) fruits, and (12) condiments. The HDDS was employed as an outcome/dependent variable in this study to demonstrate nutrition variability across market participants and non-market participants. The Kennedy cut-off points were HDDS ≤3 was considered a low dietary diversity group, with between 4–5 as medium and ≥ 6 as high diversity score category (Kennedy et al., 2011).

To assess FCS, the participants were asked to recall the foods they consumed in the previous 7 days before the survey. Each food item was given a score of 0–7 depending on the number of days it was consumed. The FCS is calculated based on the past 7-day food consumption recall for the household and classified into three categories: Poor food consumption score (0–21), borderline food consumption score (21.5–35), and acceptable food consumption score [>35; World Food Programme (WFP), 2008]. The FCS is a weighted sum of food groups. The score for each food group is calculated by multiplying the number of days the commodity was consumed and its relative weight.

The factors that affect market participation were assessed using the marginal analysis. The marginal analysis represents the residual that measures deviation from the total population mean. It is used to measure the difference from the observed value to a subject’s predicted regression. The marginal model provides crude estimates of the regression coefficients, while the conditional model has regression coefficients that are assumed common to subjects and so the estimates are adjusted for subjects.

The study’s goal is to assess the impact of market involvement on smallholder farmers’ nutrition security. It is therefore assumed that smallholder farmers who participate in the market have the potential to acquire cash and purchase more nutritious food in order to meet their daily food requirements. An investigation of the impact of treatment selection (market participation) on the outcome variable, in the jargon of impact assessment. The HDDS is the outcome variable, which is defined as the number of food groups ingested by the household in the past 24 h. Farmers’ sales and earnings can be used to define market participation. Households that participate in the market are deemed market participants and receive a score of 1, otherwise they receive a score of 0.

Treatment selection is generally influenced by subject characteristics in observational study like this. Farmers typically make voluntary decisions to enter the market based on their productive inputs and socio-demographic attributes, resulting in self-selection bias. Farmers’ market participation cannot be assigned at random in this scenario. When households are not handled randomly, their decisions for market participation can be influenced by observed and unobserved factors that correspond with the outcome variables. Another key economic obstacle in impact evaluation is the issue of missing counterfactual data. Data are missing because outcomes can only be observed in one state and counterfactuals for each group cannot be observed (Wooldridge, 2003).

Other studies (Kassie et al., 2011; Danso-Abbeam and Baiyegunhi, 2019) employed the two major econometric frameworks to address confounding factors and the issue of counterfactuals [instrumental variable (IV) and propensity scores approach]. Approaches based on propensity scores, such as regression adjustment, propensity score matching and inverse probability weighting, solely account for observed heterogeneity, whereas IV methods account for both observed and undiscovered heterogeneity. Danso-Abbeam et al. (2021) employed the instrumental variable Poisson regression model, which was also used in this study. The model estimates the causal effect of market participation on nutrition security status using the count outcome with a Poisson distribution of the error term. The primary goal of this study is to determine the average treatment effect on the treated (ATT). Takahashi and Barrett (2014) define ATT as the average difference in potential outcomes of smallholder farmers with and without market participation. The ATT can be represented as follows, according to Imbens and Wooldridge (2009) and Adolwa et al. (2019).

(1) $$ATT=E\left(Y_{1j}-Y_{0j}/T_{j}=1\right)=E\left(Y_{1j}/T_{j}=1\right)-E\left(Y_{0j}/T_{j}=1\right)$$

Where *E* {.} denotes the expectation operator, *Y*_1*j*_is the potential outcome for smallholder farmers who participate in the market, *Y*_0*j*_is the potential outcome of smallholder farmers who do not participate in the market. *T*_*j*_ Represents the treatment indicator, which takes the value 1 if smallholder farmers participate in the market and 0 otherwise. Unobserved counterfactual events pose a significant barrier in predicting the ATT. As a result, observing the prospective consequences of farmers who participated in the market if they had not participated is nearly impossible. Replacing this unobserved counterfactual with the possible results of smallholder farmers who have not engaged in the market is similarly impractical because it is likely to result in biased estimations (Takahashi and Barrett, 2014). Primary model of Terza (1998), endogenous Poisson treatment effect, is used to address the problem.

#### 2.3.1 Endogenous treatment effect model for a count outcome—Poisson

As previously stated, the study intends to see if smallholder farmers’ market participation affects their nutrition security status. Because market participation by smallholder farmers is not exogenous, it is regarded as an endogenous binary-treatment variable *T*_*j*_. *T*_*j*_ is endogenous if the treatment assignment is not random, but some unobservable covariates (variables) are affecting *T*_*j*_ that also influence the outcome variable. Since the HDDS (outcome variable) is a count event that takes values, *Y*_*j*_ = 0,1, 2,..…*Y*_*n*_ and smallholder farmers choose whether to adopt one or none, a second dummy*S*_*j*_ was developed to represent a sample selection rule. That is, smallholder farmers may not be able to engage in the market. In this case, *S*_*j*_ is missing for a proportion of the sample and the selection rule is defined such that *S*_*j*_ = 1 when *Y*_*j*_ is observed and *S*_*j*_ = 0 when *Y*_*j*_ is missing. The matter of endogeneity and sample selection was solved using the count data model with endogenous treatment (Sibhatu et al., 2015).

The Poisson endogenous treatment effect model regards the case where selection dummy *S*_*j*_ is assigned the value 0 when smallholder farmers did not receive any nutritional security status (*Y*_*j*_ is missing) and 1 when smallholder farmers did receive nutritional security status from market participation (*Y*_*j*_ is observed). Selection dummies and Endogenous treatment can be produced using continuous latent variables such as;

(2) $$T_{j}*=Z_{i}^{\prime }+\mu_{j}$$

(3) $$S_{j}^{*}=X_{j}^{\prime }\beta +\delta T_{j}+\varepsilon_{j}$$

With $T_{j}=1\left(T_{j}^{*}>0\right),S_{j}=1\left(S_{j}^{*}>0\right)$, the outcome model which follows a Poisson distribution can be specified as;

(4) $$Y_{j}=\left\{\begin{array}{l} 0\left\{\mu^{Y_{j}}\text{exp}(-\mu )\right\}/Y_{j}!\;if\;S=0\\ \;\;\;\;\;\;\;\;\;\;\;\;\;\;\;\;\;\;\;\;\;\;\;\;\;\;\;\;\;\;\;\;\;\;\;\;\;\;\;\;\;\;\;\;\;\;if\;S=1 \end{array}\right\}$$

Thus,

(5) $$E\left(Y_{j}/X_{j},T_{j},\varepsilon_{j}\right)=\text{exp}\left(X_{j}\beta +\delta T_{j}+\varepsilon_{j}\right)$$

X_*j*_ indicates the covariate vector used to model the count outcome, *Z*_*j*_ are the covariates for binary treatment, *ε*_*j*_ and *∝*_*j*_ are the error terms for the outcome and treatment, accordingly. The two error terms have a mean of zero and are bivariate normal. Since the covariates *X*_*j*_ and *Z*_*j*_ are exogenous, they are unrelated to the error terms. Conditional on *ε*_*j*_, *∝*_*j*_ is normal with mean *ε*_*jρ /σ*_ and variance (1 − *ρ*^2^). The endogenous treatment Poisson regression model is nested in a possible outcome model to estimate the ATE and ATT. The prospective outcome model describes what each farm household might receive at each treatment level.

#### 2.3.2 Ordered logistic regression model

The ordered logit regression model was used to assess the impact of determinants of market participation on the Food Consumption Score of smallholder farmers. The model was applied to perform the analysis of ordinal and categorical variables. Suppose that *h* is an ordinal dependent variable with (c) categories, and (*h* ≤ *j*) denotes the probability that the response on (H) falls in category (*j*) or below (i.e., in category 1, 2,…or j). This is called a cumulative probability. It equals the sum of the probabilities in category *j* and below:

(6) (h≤j)=(j)=Pr(h=1)+Pr(h=2)+……⋅Pr(h=j)

The category (c) and dependent (H) variable has cumulative probabilities (c): Pr (h = 1), Pr (h⩽2) … Pr (h⩽c) and the final cumulative probability uses the entire scale; therefore, Pr (h⩽c) = 1 as the order of forming the final cumulative probabilities reflects the ordering of the dependent variable’s scale, and those probabilities themselves satisfy:

(7) (h≤j)≤Pr(h=2)≤……≤(Pr(≤c))=1

An underlying probability score for an observation falling into the response category is calculated as a linear function of the independent variables and a set of cut points in an ordered logit model. The probability of observing the response category corresponds to the probability that the estimated linear function, plus a random error, falls within the range of the estimated cut points for that response.

(8) $$\begin{array}{l} \text{Pr}\;(\text{Response}\;\text{category}\;\text{for}\;\text{the}\;\text{j}\;\text{th}\;\text{outcome}\;=\text{i}\;)\;\\ \;\;\;\;\;\;\;\;\;=\text{prmi}-1<ƥ1x1j+ƥ\text{mxmj}+v\text{j}\leq \text{mj} \end{array}$$

It is important to estimate *m*_*1*_, *m*_*2*_,*…m*_*i*_ − 1, and the coefficients *ƥ*_*1*_, *ƥ*_*2*_… *ƥ*_*m*_, along with cut points *m*_*1*_, *m*_*2*_*…m*_*i*_, where (*i*) s the number of possible response categories of the dependent variable. The coefficients and cut points are estimated using a maximum likelihood Source: Own analysis. (Williams, 2006). Table 1 represents the explanatory variable that affect market participation among smallholder farmers.

## 3 Results

### 3.1 Descriptive analysis of the results

The descriptive results showed that in Mpumalanga province, only 176 farmers participated in the market and in Limpopo only 213 farmers participated in the market as shown in Table 2. Farmers who participated in the market enjoyed higher HDDS than those who did not, with an average HDDS of approximately 2 *per capita*, and those who did not participate had an average of approximately 1.89 *per capita* as shown in Table 3. This does not necessarily suggest that participation in markets can significantly improve the household food and nutrition security of rural farmers due to the selection bias issue.

The results in Tables 4, 5 represent the summary statistics of the explanatory variables used in this study. Table 4 shows the different means and standard deviations of smallholder farmers’ demographic characteristics. The results revealed that the average household age was 49.12 years, and the average household size was 4.93. The results in Table 5 indicate the variances in explanatory factors between market participants and non-market participants. In this study, 77% of the market participants were females while 23% were males. About 74% of the market participants did not have access to agricultural assistance while only 26% had access. Regarding livestock ownership, 77% of the market participants did not own any livestock while 23% had livestock. The results also showed that only 15% of market participants had access to market information, while 85% did not have access.

#### 3.1.1 Food consumption score of smallholder farmers

**Figure 2:**
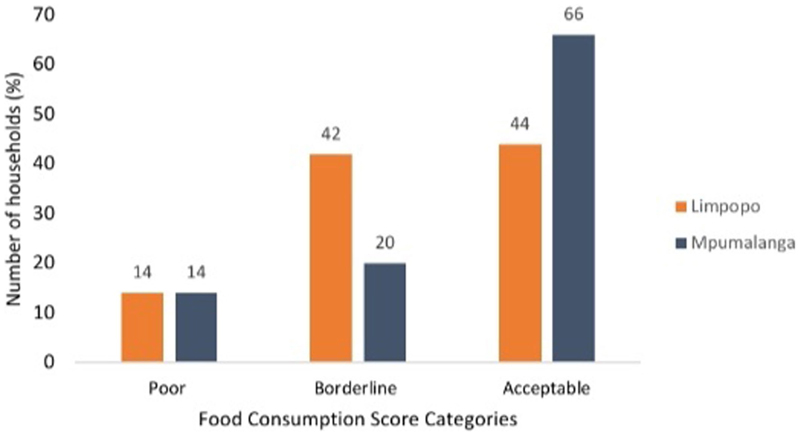
Food consumption score of smallholder farmers in Limpopo (*n* = 911) and Mpumalanga (*n* = 609) Provinces, South Africa.

The proportion of smallholder farmers’ food consumption scores before the study period is presented in Figure 2. In the overall population (*n* = 1,520), 54% of smallholder farmers were within the acceptable diversified diets, while 30 and 16% were in the borderline and poor diets, respectively. In the provinces, 44 and 66% of the smallholder farmers were consuming adequately diversified diets in Limpopo and Mpumalanga, respectively. For both provinces, 14% of the respondents consumed poor diets and this means that these farmers experienced nutrition-related problems. The results further showed that 42% (Limpopo) and 20% (Mpumalanga) of smallholder farmers were at the borderline diets and if there is no improvement in their diets they could fall into unacceptable (poor) diversity of foods.

#### 3.1.2 Dietary diversity of smallholder farmers

**Figure 3:**
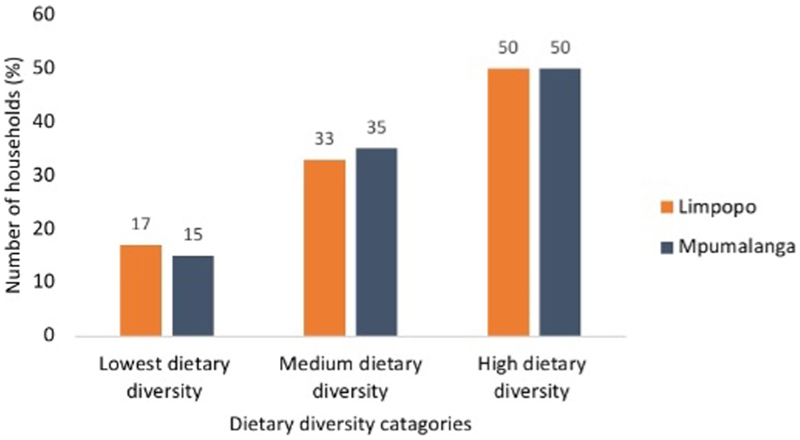
Dietary diversity of smallholder in Limpopo (*n* = 911) and Mpumalanga (*n* = 609) Provinces, South Africa.

The results in Figure 3 depict the dietary diversity of smallholder farmers in the provinces of Limpopo and Mpumalanga. Using the Kennedy et al. (2011) cut-offs, it was determined that 57% of smallholder farmers consumed highly diverse diets (more than or equal to 6 food groups), while 25 and 18% of smallholder farmers consumed medium dietary variety (between 4 and 5 food groups) and low diverse diets (less than or equal to 3 food groups), respectively. In the provinces, 50% (more or equal to 6 food groups) of smallholder farmers with highly diversified diets were found in Limpopo and Mpumalanga. In the provinces of Limpopo and Mpumalanga, smallholder consumption of a moderately diverse diet (4–5 food groups) was around 33 and 35%, respectively. While Mpumalanga had 15% of smallholder farmers, Limpopo had 17%, and both provinces had diets consisting of only three food groups or less.

### 3.2 Determinants of market participation among smallholder farmers

The factors that influenced farmers in participating in the market are presented in Table 6. The results showed that social grant, wealth index, and gender of household head all had a significant impact on the market participation of smallholder farmers. Wealth index showed a positive and statistically significant impact on the market participation of rural households; this means that an increase in wealth index of smallholder farmers increased their market participation.

The marginal effect results showed that gender of household head was statistically significant at 10%, and it had a positive coefficient. Social grant showed a significant impact on market participation of smallholder farmers and it was positive. This means that an increase in social grant led to an increase in market participation of smallholder farmers.

### 3.3 The impact of determinants of market participation on the HHDS (nutritional status) of smallholder farmers—Poisson endogenous treatment effect model

According to the Wald Chi^2^ (92.77, *p* > 0.000), the model is statistically significant at 1%, indicating a good fit. The rho (ρ) was statistically significant at 1% (0.998, *p* > 0.002). The significance of the rho *(ρ)* implies that unobserved characteristics of the smallholder farmers that influence their participation decisions in the market affect their nutritional status. To solve the issue of endogeneity, the Poisson endogenous treatment effect model should be used. The results showed that household size, agricultural assistance, ownership of livestock, social grant, wealth index, access to market information, and involvement in crop production were all statistically significant as shown in Table 7.

Household size had a positive and significant impact on the HDDS of smallholder farmers. The result showed an unexpected impact of agricultural assistance on HDDS, agricultural assistance had a negative impact on the HDDS, and it was statistically significant at 1%. Ownership of livestock had a positive and statistically significant on the HDDS of smallholder farmers. The results showed that social grant had a positive and statistically significant (*p* > 0.01) impact on the HDDS of smallholder farmers. Knowing the wealth index of a smallholder had shown a positive and significant impact. The results also showed that the involvement of smallholder farmers in crop production was statistically significant at 1% and had a positive impact on the HDDS of smallholder farmers. The coefficient on access to market information showed a positive effect on the HDDS and was significant at a 5% level.

#### 3.3.1 Treatment effects on market participation of smallholder farmers

The main focus of this study was to assess the impact of market participation on the nutrition status of smallholder farmers in terms of HDDS. Descriptive statistics results showed that the average *per capita* HDDS of smallholder farmers who participated in the market was higher than those farmers who did not participate in the market. A simple considerable difference in the average *per capita* of HDDS between market participants and non-market participants in effect assessment is misleading as it involves bias and it fails to consider the potential heterogeneity in the characteristics between the two groups. Even though it controls for endogeneity, the evaluation using the endogenous Poisson regression model may be insufficient. Because the issue of missing data (counterfactual scenario) has not been examined, direct coefficients from the model cannot be taken as Average treatment effect on the treated (ATT).

This study, therefore, turned to the results of the effects of participating in the market on the nutrition status of smallholder farmers in terms of HDDS using ATT and ATE, where the Poisson regression with endogenous treatment effects was used. The ATE and ATT were assessed after fitting the Poisson regression with endogenous treatment effects. As shown in Table 8, the estimated potential outcome means (ATE) of market participation on HDDS was 0.747 and was statistically significant at 1%. The ATE estimate indicated that the average smallholder farmers who participated in the market in the whole sampled population had improved nutritional status. Correspondingly, the conditional treatment effect, which measures the ATT of market participation on HDDS, was 0.768 and statistically significant at 1%. Therefore, smallholder farmers who participated in the market had an average of 0.768 more of HDDS than it would if they did not participate in the market.

### 3.4 The impact of determinants of market participation on the food consumption score of smallholder farmers—ordered logistic regression model

The results in Table 9 indicate the impact of market participation on the food consumption score of smallholder farmers. The result showed that household size had a negative and significant impact on the food consumption score of smallholder farmers. Gender of household head, irrigation type, social grant, and amount harvested had a positive and significant impact on the food consumption score of smallholder farmers.

## 4 Discussion

Food consumption score and dietary diversity are dominant topics in the scientific world of nutrition because they serve as accelerators for enhanced nutrition-sensitive and customized programs by recognizing nutrient deficiencies in families (Ambaw et al., 2021). According to the literature, socio-demographics, knowledge, attitude, and household assets are some of the essential components connected with the level of food consumption score among households (Daba et al., 2013; Ambaw et al., 2021). Primarily, unacceptable food consumption score is the main public health problem, and thus, strengthening nutrition intervention is very important (Isaura et al., 2018). The current study found that slightly more than half (54 and 57%) smallholder farmers had acceptable food consumption scores and highest dietary diversity, respectively. These results were in line with that of Fite et al. (2022), who discovered that more than half of the pregnant women in Haramaya District, eastern Ethiopia had acceptable food consumption score. The study indicated that factors such as consumption of animal-source foods, attitude, wealth and agricultural land possession were positively associated with acceptable food consumption score.

Household size refers to the total number of family members who live and eat in the same house for at least 6 months (Muche et al., 2014). According to Mequanent (2009) and Beyene and Muche (2010), household size or family size is an important factor that affects the state of household food security and, in most cases, has a negative impact on household food security. The influence of household size varies depending on the type of household and the density of the household size. In this study, household size had a positive impact on smallholder farmers’ dietary diversity (HDDS). This suggests that the majority of households were old enough to participate in the production and the market. The findings of this study, however, revealed that household size had a negative impact on the food consumption score of smallholder farmers. One probable explanation is that as family sizes grow, people compete for the little food they have and end up consuming fewer portions of various foods. Tsegay (2009) confirmed this, reporting that increasing family size puts more pressure on food consumption than on labor for production. Furthermore, Muche et al. (2014) reported that large family sizes put more pressure on household food security because it led to more food and non-food expenditures.

The accessibility of agricultural assistance had a detrimental impact on the HDDS. A possible explanation for this is that not all smallholder farmers benefit from government extension services, which prevents them from producing enough to meet market demand. Most smallholder farmers produce mostly staple crops using whatever resources they have and traditional ways. However, the findings contradict those of Fischer and Qaim (2012), Jari and Fraser (2012), Kyaw et al. (2018) and Sebatta et al. (2014), who discovered a positive and substantial relationship. According to these studies, having access to agricultural support can provide information about market access and improved varieties, which can increase farmers’ production knowledge. It can also provide farmers with a variety of seeds to enable them to produce a diverse range of crops for sale and consumption.

Livestock is an important production shifter since it increases a household’s potential to produce more, boosting the likelihood of a household’s market participation (Pica-Ciamarra et al., 2011). The findings of this study revealed that livestock ownership had a favorable impact on the HDDS of smallholder farmers. Smallholder farmers with livestock can sell some of their livestock to purchase nutritious foods while simultaneously investing more in crop cultivation. However, Kyaw et al. (2018) noted that if a household owns livestock, the household members would need to split time and money with the livestock for feeding and caring for the livestock, resulting in less production surplus to sell in the market. The study further explained that farmers with insufficient land must sacrifice crop output and focus on livestock rearing, which may have a detrimental impact on their marketable surplus.

Social grants have grown in popularity as a tool of enhancing the well-being of impoverished households in South Africa and beyond (Grinspun, 2016). The findings of this study demonstrated that the social grant had a favorable impact on smallholder farmers’ market participation, FCS, and HDDS. This is due to the fact that the purpose of social grants is to alleviate poverty and increase human capital capacity. Many studies have found that social grants can boost rural households’ economic capacity; yet many rural households misuse social grant funds (Covarrubias and Davis, 2012; Boone et al., 2013; Sinyolo et al., 2017; Todd et al., 2020). According to these studies, the majority of households rely solely on social grants and ignore alternative activities that can help them generate revenue. This resulted in them consuming lower-quality food and affecting their dietary diversity.

Smallholder farmers that know the resources they possess and their living standards, they tend to utilize what they have and produce effectively. Our results confirmed that high wealth index had a positive impact on market participation and nutrition status of smallholder farmers. The results also showed that the involvement of smallholder farmers in crop production had a positive impact on the HDDS of smallholder farmers. This was because smallholder farmers who are involved in crop production use the opportunity of market participation by being both sellers and buyers. They produce more variety of crops to sell the surplus in the market and they also use the income earned to buy other food groups that they cannot produce. This result is in line with Mathenge et al. (2010) and Mulenga et al. (2021) who reported that farmers who are involved in crop production have more comparative advantages in resource use, which can be shown in improved productivity through economies of scale.

The positive outcome of market information implies that farmers who have access to market information are likely to sell their products and make a profit. The results showed that the market information helped farmers with the knowledge of the market. Farmers were able to get information on pricing strategies and information on the crops that are in demand. This result is similar to that of Kyaw et al. (2018) who reported that access to market information would lead to increased productivity with a high marketable surplus. Moreover, Irrigation type and amount of crop harvested showed positive results on the FSC of smallholder farmers. This is because having access to irrigation system make smallholder farmers to depend less on rain and be able to produce crops under unfavorable weather conditions. In contrary to this, Fanadzo et al. (2010) and Post et al. (2012) reported that majority of smallholder farmers in rural areas do have access to irrigation system. These studies further reported that most of the smallholder farmers depend on rainfall for their production which is why they less diversity their crop production.

## 5 Limitations of the study and directions for future research

The data used in this analysis were gathered throughout the 2016/2017 season. Smallholder farmers’ farming and marketing systems may have evolved since then. To understand how the situation has changed since the last data collection, recent primary data are required. This study concentrated on two South African provinces. There is a need to broaden research to include all provinces, as well as comparing market involvement across provinces to draw lessons from one to the next province.

## 6 Conclusion and recommendations

The magnitude of under nutrition in developing countries remains high despite improvements in food and nutrition security over the last few decades. The results showed that more than half of smallholder farmers had acceptable food consumption score and highest dietary diversity. Therefore, this study recommends that more specific nutrition programs should be established to improve the nutritional status of smallholder farmers. The result also showed that agricultural assistance had a significant impact on the HDDS of smallholder farmers. The improvement of agricultural assistance is more associated with improvement of extension services, which can lead to more production of diverse crops and more market participation.

Health extension workers need to do more nutrition programs and workshops in rural areas. These programs and workshops will be intended on providing nutrition education, which will create awareness to smallholder farmers on diverse and balanced food items they should produce, sell, and consume. The extension workers can also invite nutritional counselors that will address the importance of food diversity. The nutritional counselor helps by demonstrating and providing guidebooks on all the diverse and balanced food items required (Ambaw et al., 2021). The workshops will also be used to help smallholder farmers on how they can improve their market participation. The extension workers need to come up with different and easy strategies on delivering market information to smallholder farmers. Hudson et al. (2017) suggested that extension workers need to use more participatory approaches in order to deliver knowledge to smallholder farmers since most of them are uneducated. This will help smallholder farmers in making production decisions that are in line with consumers’ demand.

## Figures and Tables

**Table 1:** *A priori* expectations for the explanatory variables used in the models.

Variables names	Variable type and measurement
Age of the household head	Participant’s age in years.
Gender of household head	If the respondent is male, 1; otherwise, 0.
Marital status	If the participant is married, 1 is assigned; otherwise, 0 is assigned.
Household size	The farm household’s total family members.
Education level of the household head	Years of education (continuous).
Ownership of livestock	If the participant owned livestock, 1; otherwise, 0.
Access to market information	1 if participants received market information, 0 otherwise
Involvement in crop production	0 if respondents were not active in crop production, 1 if they did.
Disability in the family	If there is a disabled member of the family, 1 is assigned; otherwise, 0 is assigned.
Access to agricultural assistance	If interviewees have access to extension services, they received a 1; otherwise, they received a 0.
Family member with HIV	If there is an HIV-positive family member, 1; otherwise, 0.
Family member worked on farm	If there is a family member who worked on the farm, 1; otherwise, 0.
Income	If there is a person who works for income, 1; otherwise, 0.
Social grant	If a family member receives a social grant, 1 is assigned; otherwise, 0 is assigned.
Irrigation type	If the participant had access to an irrigation system, the answer was 1, otherwise it was 0.

Source: Own analysis.

**Table 2:** The extent of market participation of smallholder farmers in South Africa’s Limpopo and Mpumalanga provinces.

		Market participants	Non-market participants	Total
Province name	Mpumalanga	176	433	609
	Limpopo	213	698	911
Total		389	1,131	1,520

Source: Own analysis.

**Table 3:** The difference HDDS between market participant and non-market participant.

Variables	Mean	Standard deviation
Household dietary diversity (HDDS)		
Market participant	2.134	1.982
Non-market participant	1.982	1.218

Source: Own analysis.

**Table 4:** Demographic characteristics of smallholder farmers in Limpopo and Mpumalanga provinces, South Africa.

Variable	Mean ± Standard Deviation (SD)
Household age	49.12 ± 11.89
Household size	4.93 ± 2.71
Educational level of household	33.58 ± 40.30
Ownership Livestock	1.77 ± 0.42
Distance to the market	1.86 ± 1.82
Family member with HIV	0.47 ± 0.79
Family member worked on a farm	0.98 ± 0.76
Social grant	1.99 ± 0.73

Source: Authors’ own analysis.

**Table 5:** Demographic characteristics of smallholder farmers in Limpopo and Mpumalanga provinces, South Africa.

Variable	Market participant (*n*= 389)		Non-market participant (*n*= 1,131)		Overall Freq
	%	Freq	%	Freq	
Gender of household					
Female	77	300	61	688	988
Male	23	89	39	443	532
Access to agricultural assistance					
Yes	26	100	28	318	418
No	74	289	72	813	1,102
Access to market information					
Yes	15	60	34	387	447
No	85	329	66	744	1,073
Ownership of livestock					
Yes	23	89	37	414	503
No	77	300	63	717	1,017

Source: Authors’ own analysis.

**Table 6:** Factors influencing market participation among smallholder farmers.

Market participation	Probit			Marginal effect		
	Coeff	St.Err.	*p* value	dy/dx	St.Err.	*p* value
Household size	0.032	0.045	0.476	0.001	0.001	0.477
Gender of household head (male = 1, 0 otherwise)	0.644	0.319	0.043**	0.015	0.008	0.053*
Age of household head	−0.004	0.008	0.599	−0.000	0.000	0.600
Educational level of household head	−0.258	0.426	0.546	−0.006	0.010	0.545
Marital status of household head (married =1, 0 otherwise)	−0.151	0.452	0.739	−0.004	0.011	0.739
Agricultural assistance	0.235	0.423	0.566	−0.002	0.011	0.543
Family member with HIV	−1.222	0.473	0.465	−0.029	0.011	0.445
Social grant	1.184	0.335	0.000***	0.028	0.008	0.001***
Wealth index	1.021	0.163	0.000***	0.024	0.005	0.000***
Amount harvested	0.000	0.001	0.785	−0.000	0.000	0.785
Constant	0.509	0.798	0.524			
Mean dependent var	0.649					
Pseudo *r*-squared	0.926					
Chi-square	1268.316					
Akaike crit. (AIC)	120.816					
Prob > chi^2^	0.000					
Bayesian crit. (BIC)	170.439					

Dependent variable is market participation; ***, **, and * indicate significance at 1, 5, and 10% level, respectively. Source: Own analysis.

**Table 7:** Determinants of nutrition status using Poisson regression with endogenous treatment.

Variables	Coef.	Std.Err.	*p* value
HDDS			
Age of the household head	−0.000	0.001	0.583
Gender of household head	−0.009	0.022	0.676
Households size	0.009	0.002	0.000***
Educational level of household head	−0.047	0.054	0.391
Marital status	0.023	0.054	0.667
Access to agricultural assistance	−0.090	0.014	0.000***
Ownership of livestock	0.123	0.057	0.030**
Family member worked for a wage salary	0.009	0.043	0.838
Social grant	0.038	0.020	0.056*
WEATHINDEX	0.058	0.024	0.015**
Access to market information	−0.038	0.018	0.031**
Involvement in crop production	0.199	0.058	0.001***
Family member with HIV	0.004	0.052	0.687
Market participation	0.084	0.029	−2.950
_cons	2.066	0.095	21.730
Market participation			
If household received agricultural related assistance	2.592	0.028	93.210
_constant	−0.931	0.015	−62.570
/athrho	3.430	0.374	9.170
/lnsigma	−17.326	0.107	−161.710
Wald Chi^2^ (15)	92.77 0.000		
rho *(ρ)*	0.998	0.002	
sigma *(σ)*	0.000	0. 000	

Dependent variable is HDDS; ***, **, and * indicate significance at 1, 5, and 10% level, respectively. Source: Authors’ own analysis.

**Table 8:** Treatment effects on market participation of smallholder farmers.

Treatment effects	Coefficient	Std.Err.	*p* value
*Poisson regression* *with* *treatment* *effects*			
Average treatment (ATE)	0.747	0.267	0.003***
Average treatment effect on the treated (ATT)	0.768	0.255	0.004***

***, **, and * indicate significance at 1, 5, and 10% level, Source: Authors’ own analysis.

**Table 9:** Determinants of food consumption scores using ordered logistic regression model.

Variables	Coef.	Std.Err.	*p* value
Food consumption scores			
Age of household head	−0.036	0.081	0.654
Household size	−0.058	0.031	0.063*
Gender of household head	0.874	0.305	0.004***
Educational level of household head	1.167	0.902	0.196
Irrigation type	0.947	0.501	0.059*
Marital status	1.073	0.676	0.112
Main economic activity	−0.853	0.326	0.123
Family member with HIV	−0.739	0.599	0.217
Distance to the market	0.943	0.618	0.127
Social grant	−0.805	0.242	0.001***
Amount harvested	0.001	0.000	0.068*
Cut 1	1.749	4.312	. b
Cut 2	3.470	4.316	. b
Cut 3	7.693	4.434	. b
Mean dependent var	1.365	SD dependent var.	0.620
Pseudo *r*-squared	0.032	Number of obs	788.000
Chi-square	38.909	Prob > chi^2^	0.000
Akaike crit. (AIC)	1212.228	Bayesian crit. (BIC)	1286.940

Dependent variable is FCS; ***, **, and * indicate significance at 1, 5, and 10% level, respectively. Source: Authors’ own analysis.

## Data Availability

The data analyzed in this study is subject to the following licenses/restrictions: restriction apply to the availability of these data. Data were obtained from the Department of Agriculture, Land Reform, and Rural Development (DALRRD) and are available from South African Vulnerability Assessment Committee (SAVAC) secretariat with the permission of Department of Agriculture, Land Reform, and Rural Development (DALRRD). Requests to access these datasets should be directed to www.dalrrd.gov.za.

## References

[R1] Adeyeye SAO, Ashaolu TJ, Bolaji OT, Abegunde TA, Omoyajowo AO (2021). Africa and the Nexus of poverty, malnutrition and diseases. Crit Rev Food Sci Nutr.

[R2] Adolwa IS, Schwarze S, Buerkert A (2019). Impacts of integrated soil fertility management on yield and household income: the case of tamale (Ghana) and Kakamega (Kenya). Ecol Econ.

[R3] Akombi BJ, Agho KE, Merom D, Renzaho AM, Hall JJ (2017). Child malnutrition in sub-Saharan Africa: a meta-analysis of demographic and health surveys (2006-2016). PloS One.

[R4] Ambaw MB, Shitaye G, Taddele M, Aderaw Z (2021). Level of food consumption score and associated factors among pregnant women at SHEGAW MOTTA hospital, Northwest Ethiopia. BMC Public Health.

[R5] Baird S, Hicks JH, Kremer M, Miguel E (2016). Worms at work: long-run impacts of a child health investment. Q J Econ.

[R6] Bellon MR, Ntandou-Bouzitou GD, Caracciolo F (2016). On-farm diversity and market participation are positively associated with dietary diversity of rural mothers in southern Benin, West Africa. PloS One.

[R7] Beyene F, Muche M (2010). Determinants of food security among rural households of Central Ethiopia: an empirical analysis. Q J Int Agric.

[R8] Boone R, Covarrubias K, Davis B, Winters P (2013). Cash transfer programs and agricultural production: the case of Malawi. Agric Econ.

[R9] Covarrubias K, Davis B (2012). Do unconditional social cash transfer schemes have productive impacts in Malawi. Int Policy Center Inclus Growth.

[R10] Daba G, Beyene F, Fekadu H, Garoma W (2013). Assessment of knowledge of pregnant mothers on maternal nutrition and associated factors in Guto Gida Woreda, east Wollega zone, Ethiopia. J Nutr Food Sci.

[R11] Danso-Abbeam G, Baiyegunhi LJ (2019). Does fertiliser use improve household welfare? Evidence from Ghana’s cocoa industry. Dev Pract.

[R12] Danso-Abbeam G, Ojo TO, Baiyegunhi LJ, Ogundeji AA (2021). Climate change adaptation strategies by smallholder farmers in Nigeria: does non-farm employment play any role?. Heliyon.

[R13] De Cock N, D’Haese M, Vink N, Van Rooyen CJ, Staelens L, Schönfeldt HC (2013). Food security in rural areas of Limpopo province, South Africa. Food Secur.

[R14] Drammeh W, Hamid NA, Rohana AJ (2019). Determinants of household food insecurity and its association with child malnutrition in sub-Saharan Africa: a review of the literature. Curr Res Nutr Food Sci J.

[R15] Fanadzo M, Chiduza C, Mnkeni PNS (2010). Overview of smallholder irrigation schemes in South Africa: relationship between farmer crop management practices and performance. Afr J Agric Res.

[R16] Fischer E, Qaim M (2012). Linking smallholders to markets: determinants and impacts of farmer collective action in Kenya. World Dev.

[R17] Fite MB, Tura AK, Yadeta TA, Oljira L, Roba KT (2022). Factors associated with food consumption score among pregnant women in eastern Ethiopia: a community-based study. J Health Popul Nutr.

[R18] Getahun T, Fetene G (2021). The Nexus of production diversity, market participation and dietary diversity: insights from Ethiopia. ZEF Discuss Papers Dev Policy.

[R19] Grinspun A (2016). No small change: the multiple impacts of the child support Grant on child and adolescent well-being. Child Gauge.

[R20] Hirvonen K (2016). Rural–urban differences in children’s dietary diversity in Ethiopia: a Poisson decomposition analysis. Econ Lett.

[R21] Hudson HE, Leclair M, Pelletier B, Sullivan B (2017). Using radio and interactive ICTs to improve food security among smallholder farmers in sub-Saharan Africa. Telecommun Policy.

[R22] Imbens GW, Wooldridge JM (2009). Recent developments in the econometrics of program evaluation. J Econ Lit.

[R23] International Food Policy Research Institute (FPRI) (2014). Global nutrition report 2014: Actions and accountability to accelerate the World’s Progress on nutrition.

[R24] Isaura ER, Chen YC, Yang SH, Schalkwyk Herman D, Van Groenewald JA (2018). The association of food consumption scores, body shape index, and hypertension in a seven-year follow-up among Indonesian adults: a longitudinal study. Int J Environ Res Public Health.

[R25] Jari B, Fraser G, Schalkwyk Herman D, Van Groenewald JA (2012). Influence of Institutional and Technical Factors on Market Choices of Smallholder Farmers in the Kat River Valley.

[R26] Kassie M, Shiferaw B, Muricho G (2011). Agricultural technology, crop income, and poverty alleviation in Uganda. World Dev.

[R27] Kennedy G, Ballard T, Dop MC (2011). Guidelines for measuring household and individual dietary diversity. Food and Agriculture Organization of the United Nations.

[R28] Kyaw NN, Ahn S, Lee SH (2018). Analysis of the factors influencing market participation among smallholder rice farmers in Magway Region, central dry zone of Myanmar. Sustain For.

[R29] Lenjiso BM, Smits J, Ruben R (2016). Smallholder milk market participation, dietary diversity and nutritional status among young children in Ethiopia. J Gender Agric Food Secur.

[R30] Manyamba C, Hendriks S, Chilonda P, Musaba E (2012). Factors contributing to inequalities in food security in South Africa: implications for agricultural policy.

[R31] Mathenge M, Place F, Olwande J, Mithoefer D (2010). Participation in agricultural markets among the poor and marginalized: analysis of factors influencing participation and impacts on income and poverty in Kenya.

[R32] Mequanent M (2009). Determinants of household food security and coping strategy: The case of Adaberga Woreda, west Shoa zone, Ethiopia.

[R33] Mpumalanga Department of Finance (2013). Socio-economic review and outlook of Mpumalanga.

[R34] Muche M, Endalew B, Koricho T (2014). Determinants of household food security among Southwest Ethiopia rural households. Food Sci Technol.

[R35] Mulenga BP, Ngoma H, Nkonde C (2021). Produce to eat or sell: panel data structural equation modeling of market participation and food dietary diversity in Zambia. Food Policy.

[R36] NAFCOC (2014). Provinces:Mpumalanga.

[R37] Obi A, Schalkwyk HDV, Tilburg AV, Schalkwyk HDV, Groenewald JA, Fraser GCG, Obi A, Tilburg AV (2012). Unlocking Markets to Smallholders.

[R38] Pica-Ciamarra U, Tasciotti L, Otte J, Zezza A (2011). Livestock assets, livestock income and rural households: cross-country evidence from household surveys.

[R39] Post DA, Chiew FHS, Teng J, Viney NR, Ling FLN, Harrington G (2012). A robust methodology for conducting large-scale assessments of current and future water availability and use: a case study in Tasmania, Australia. J Hydrol.

[R40] Sebatta C, Mugisha J, Katungi E, Kashaaru A, Kyomugisha H (2014). Smallholder farmers’ decision and level of participation in the potato market in Uganda. Mod Econ.

[R41] Sibhatu KT, Krishna VV, Qaim M (2015). Production diversity and dietary diversity in smallholder farm households. Proc Natl Acad Sci.

[R42] Sinyolo S, Mudhara M, Wale E (2017). The impact of social grant-dependency on agricultural entrepreneurship among rural households in KwaZulu-Natal, South Africa. J Dev Areas.

[R43] Ssajakambwe F, Mulebeke R, Elepu G, Walekhwa PN (2020). Linking market access to improved nutrition among smallholder maize farmers in Masindi and Kiryandongo districts, Uganda. Afric J Market Manag.

[R44] Statistics South Africa (2015). Limpopo community survey 2016 results.

[R45] Statistics South Africa (2022). Focus on food inadequacy and hunger in South Africa in 2021.

[R46] Swindale A, Bilinsky P (2006). Development of a universally applicable household food insecurity measurement tool: process, current status, and outstanding issues. J Nutr.

[R47] Takahashi K, Barrett CB (2014). The system of rice intensification and its impacts on household income and child schooling: evidence from rural Indonesia. Am J Agric Econ.

[R48] Terza JV (1998). Estimating count data models with endogenous switching: sample selection and endogenous treatment effects. J Econ.

[R49] Todd JE, Winters PC, Hertz T, Carletto C, Davis B, Winters P (2020). Migration, Transfers and Economic Decision Making among Agricultural Households.

[R50] Tsegay G (2009). Determinants of food security in rural households of the Tigray region, 2009.

[R51] United Nations International Children’s Emergency Funds (UNICEF) (2022). The state of food security and nutrition in the world 2022—UNICEF DATA.

[R52] Williams R (2006). Generalized ordered logit/partial proportional odds models for ordinal dependent variables. Stata J.

[R53] Wooldridge JM (2003). Cluster-sample methods in applied econometrics. Am Econ Rev.

[R54] World Food Programme (WFP) (2008). Food consumption score vulnerability assessment and mapping.

